# Accelerating Photochemical Insight with “Roblonski”, a Robo-Fluidic Platform

**DOI:** 10.1021/acscentsci.6c00446

**Published:** 2026-04-29

**Authors:** Reem Nsouli, Gaurav Galiyan, Laura K. G. Ackerman-Biegasiewicz

**Affiliations:** † Department of Chemistry, 1371 Emory University, Atlanta, Georgia 30322, United States; ‡ School of Molecular Sciences, 7864 Arizona State University, Tempe, Arizona 85287, United States

## Abstract

A robo-fluidic platform enables automated photophysical measurements for cost- and time-efficient photochemical characterization.

## Graphical abstract

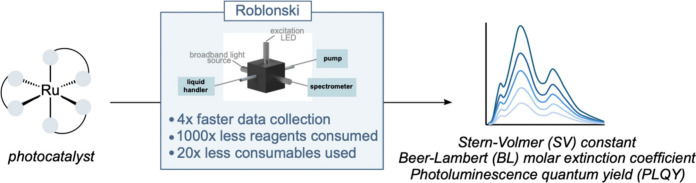

Over the past two decades, photocatalysis has matured into a central tool in modern synthesis, enabling new bond constructions, expanding redox reactivity, and providing sustainable routes across a wide range of applications.^1,2^ However, quantitative photophysical characterizations, which are essential for mechanistic interpretation, remain disproportionately laborious relative to reaction screening workflows.^3^ Conventional workflows, often requiring sequential and manual sampling and dilutions, are resource- and time-intensive, which limits the throughput and reproducibility of these foundational analyses. An alternative paradigm can be envisioned that takes advantage of automation in which photophysical assays are miniaturized and executed in minutes’ time through robotic handling. That is the promise of a new robo-fluidic platform designed to transform how researchers characterize photochemical reactions.

In a recent issue of *ACS Central Science*, Castellano, Abolhasani, and co-workers introduced “Roblonski”, a compact robo-fluidic platform designed to streamline and automate photochemical analyses (Figure 1a).^4^ The platform integrates rapid Stern–Volmer (SV) analysis, Beer–Lambert (BL) measurements, and photoluminescence quantum yield (PLQY) determination within a unified and resource-efficient framework. Architecturally, Roblonski employs a pipet-based robotic liquid handler interfaced with a microfluidic flow cell which is coupled to a broadband light source, an excitation LED, and a spectrometer for spectral acquisition. This system combines batch-style serial dilutions with a microfluidic flow-based detection interface, enabling precise sample preparation and rapid data collection within a single automated workflow (Figure 1b).^5–7^

**Figure 1:**
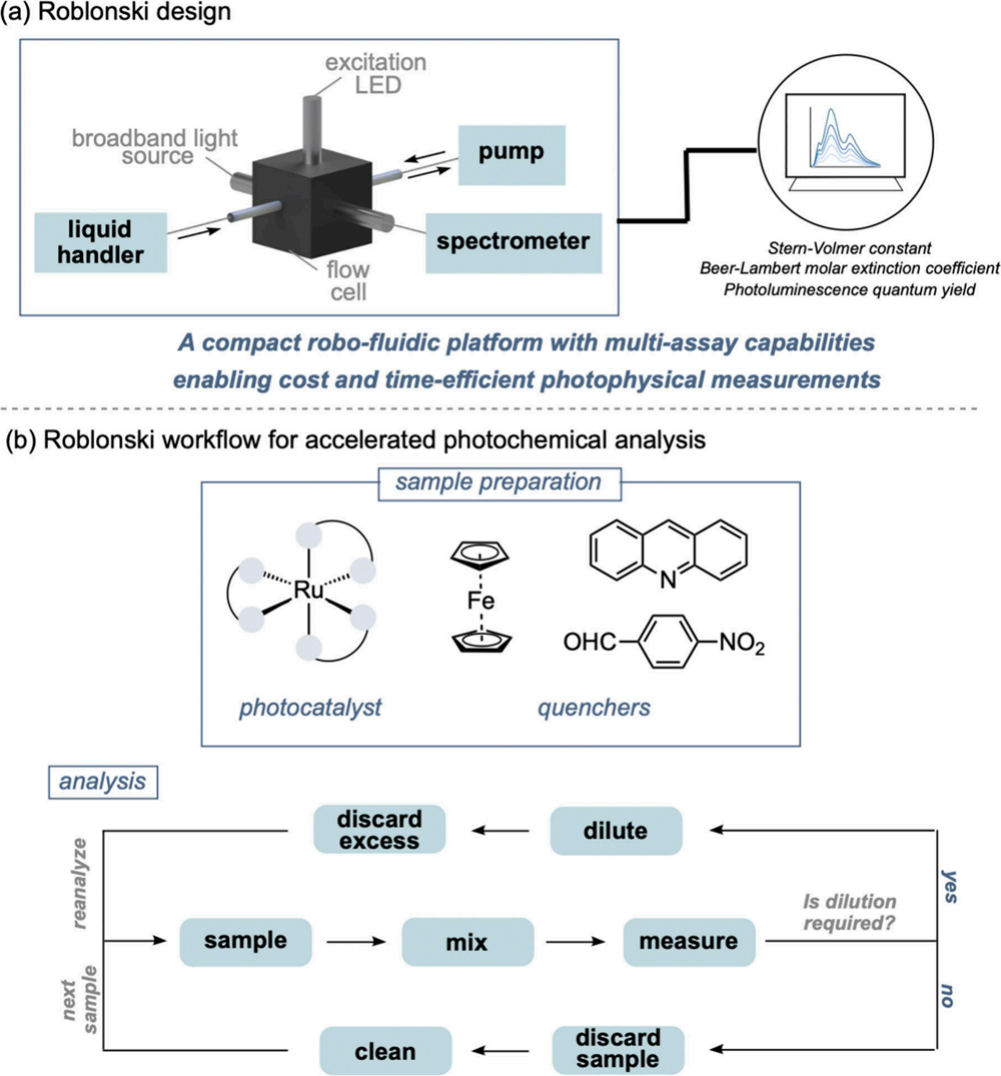
(a) Illustration of the robo-fluidic platform, Roblonski, and its photoanalytical capabilities. (b) Workflow and benchmarking of Roblonski for automated dilution and analysis.

Unlike prior automation efforts that focus on a single assay modality, Roblonski consolidates multiple foundational measurements into a small-footprint, cost-conscious, and material-efficient platform.

The authors benchmark the platform rigorously against conventional manual, cuvette-based measurements and established literature results. Using Ru­(bpy)_3_(PF_6_)_2_ and 11 diverse quenchers, SV studies were conducted in which quenching rate constants across oxidative electron transfer, reductive electron transfer, and energy transfer regimes were determined with high accuracy. Additionally, comparable agreement was observed for BL analyses of five compounds exhibiting diverse electronic transitions and a broad range of molar extinction coefficients in various solvents. The authors further demonstrate consistency for PLQY measurements of five compounds ranging from weakly emissive metal complexes to highly fluorescent organic chromophores. Collectively, these results established a high level of reproducibility, precision, and accuracy across diverse spectral profiles and mechanistic regimes. When comparing a manual cuvette approach with the Roblonski automated approach, it was determined that Roblonski was four times faster for data acquisition while consuming 1000 times less reagent and 20 times fewer consumable materials such as pipet tips.

The conceptual significance of this work lies not only in reducing acquisition time, material consumption, and reagent volume but also in repositioning photophysical characterization as a scalable infrastructure. Typically, SV analysis and PLQY determination are performed selectively and often only after promising results have been identified. By lowering the barrier to photophysical studies, Roblonski enables systematic examination of catalyst–substrate interactions and a comprehensive analysis of photochemical systems. Additionally, the automation of these measurements provides a platform for fast and reliable library generation. This data density is critical for benchmarking new catalysts and correlating excited-state properties with catalytic performance, ultimately leading to enhanced understanding and rapid reaction discovery.

While Roblonski represents a clear step forward in automated photophysical characterization, this work also highlights areas where further refinement may prove valuable as the platform evolves. Like many flow-based analytical systems, performance remains dependent on solubility and phase homogeneity, whereby precipitation may affect both liquid handling and spectral fidelity. In addition, the operation, while automated, is not fully autonomous, requiring users to initially define concentration ranges, signal thresholds, and acquisition parameters. Furthermore, expansion to new assay types or spectral domains beyond UV–vis spectroscopy would require hardware modification, and increasing functionality would inevitably introduce additional complexity and cost. As with many modular automation platforms, the balance between breadth of capability and economic accessibility remains an important consideration for widespread adoption.

With Roblonski’s core architecture established, this platform provides a foundation for further expansion in functionality and integration. One significant avenue of interest is expanding the system to enable time-resolved measurements that directly probe excited-state lifetimes and intermediate formation, providing meaningful mechanistic insights. Furthermore, integration with laboratory-assistance or experiment-planning software, such as Coscientist,^8^ could further reduce operator burden and improve accessibility and user-friendliness. The authors also suggest the addition of temperature control within the liquid-handling or microfluidic module to improve solubility and enable microscale synthesis and characterization under controlled conditions.

Overall, this platform aligns with a broader movement toward automated infrastructures for photochemistry.

By integrating this workflow with high-throughput experimentation infrastructure, Roblonski provides a modular framework that could interface naturally with reaction optimization platforms or machine-learning guided discovery pipelines.
